# 

**Published:** 1865-02

**Authors:** 


### CONTENTS.

### ORIGINAL CONTRIBUTIONS.

ART. IV. Climate and Diseases of New Mexico. Remarkable Exemption from Consumption. Prevalence of Rheumatism and Syphilis, .......... 65
ART. V. Rubeola Complicated with Pneumonia. By P. J. Wardner, M.D., of Springfield, Ill., .......... 77
ART. VI. Alcoholic Stimulants in Typhus and Typhoid Fevers. By the Editor, .......... 82
Correspondence from New York. By R. P. J., .......... 79

### SELECTIONS.

On the Prevention of Zymotic and Constitutional Diseases: Fevers; Cholera; Consumption, &c. By Dr. E. D. Mapother, Prof., &c., .......... 84
On Epidemic Cerebro-Spinal Meningitis, or Spotted Fever, with Cases. By John A. Liddell, M.D., Surgeon U.S.V., .......... 90
Two Cases of Pyæmia, or Purulent Infection, with Recovery; in which the Bisulphite of Soda was Administered. Communicated by Walter F. Atlee, M.D., .......... 105
On the Transformation of Alkaline Sulphites in the Human System. By M. Carey Lea, .......... 108
Permanganate of Potash as a Remedy for Diphtheria. By Lewis Mackall, Jr., M.D., Georgetown, D.C., .......... 111

### BOOK NOTICES.

Transactions of the American Medical Association. Vol. XV. .......... 113
Medical Lexicon: A Dictionary of Medical Science, .......... 115
Gun-Shot Wounds and other Injuries of Nerves, .......... 115
Glaucoma: Its Symptoms, Diagnosis, and Treatment, .......... 116

### EDITORIAL.

Notice, .......... 116
Chicago Medical College. Sixth Annual Commencement, .......... 117
Chicago Medical Society, .......... 118
Mortality of Chicago for February, 1865, .......... 119
New Hospital for Women and Children, .......... 120
Personal. Dr. H. A. Johnson, .......... 121
Hospital Appointment. Dr. J. S. Jewell, .......... 121
Illinois State Medical Society, .......... 121
American Medical Association, .......... 122
Destruction of Tumors by Electricity, .......... 123
Army Medical Intelligence, .......... 123
Effect of Petroleum on the Nervous System, .......... 123
Renal Calculi, .......... 123
Existence in the Human Subject of Organs unprovided with Nerves, Lymphatics, or Capillaries, .......... 124
Deformities of the Feet and the Treatment by Means of Plaster Paris, .......... 125

